# Pleuropulmonary Epithelioid Hemangioendothelioma

**DOI:** 10.7759/cureus.69967

**Published:** 2024-09-23

**Authors:** Philippe Haroun, Hanna Salame, Denis Tack, Romain Dehon

**Affiliations:** 1 Radiology, Université Libre de Bruxelles (ULB), Brussels, BEL; 2 Radiology, Centre Hospitalier EpiCURA - Site de Hornu, Hornu, BEL; 3 Radiology, Centre Hospitalier EpiCURA - Site de Ath, Ath, BEL; 4 Pathology, Institute of Pathology and Genetics, Charleroi, BEL

**Keywords:** ct chest, epitheloid hemangioendothelioma, pleural epithelioid hemangioendothelioma, pleuropulmonary, video-assisted-thoracoscopy

## Abstract

We present the CT, thoracoscopic, and histological findings of a 55-year-old female with pleuropulmonary epithelioid hemangioendothelioma. The CT scan revealed pleuropulmonary lesions, and thoracoscopy allowed direct visualization and biopsy of the lesions. Histological analysis confirmed the diagnosis, emphasizing the utility of combining imaging, endoscopic, and pathological methods in diagnosing this rare tumor.

## Introduction

Epithelioid hemangioendothelioma (EHE) are uncommon low-to-intermediate-grade vascular tumors originating from the endothelium [1]. EHEs can involve various organs, including the lung, liver, brain, bone, soft tissue, and breast [1,2]. These tumors lack specific radiologic features and have no specific biological markers. Therefore, diagnosis of EHE relies on pathologic examination of specimens obtained by surgical biopsy [3]. Immunohistochemistry is required to determine the vascular endothelial cell phenotype, which consists of the expression of markers such as CD31, ERG, CD34, and FLI-1 [1,2]. Weiss and Brighe coined the term “malignant” EHE for tumors that exhibit marked nuclear atypia, mitotic activity (>1 mitoses per 10 HPF), tumor necrosis, or spindle cell component [4]. We hereby present the case of a patient manifesting features of pulmonary and pleural EHE.

## Case presentation

A 55-year-old woman presented to the emergency department with progressive shortness of breath and cough. The patient denied any history of smoking and asbestos exposure. Anamnesis revealed that 25 years ago, the patient had a diagnosis of pulmonary and osseous EHE.

**Figure 1:**
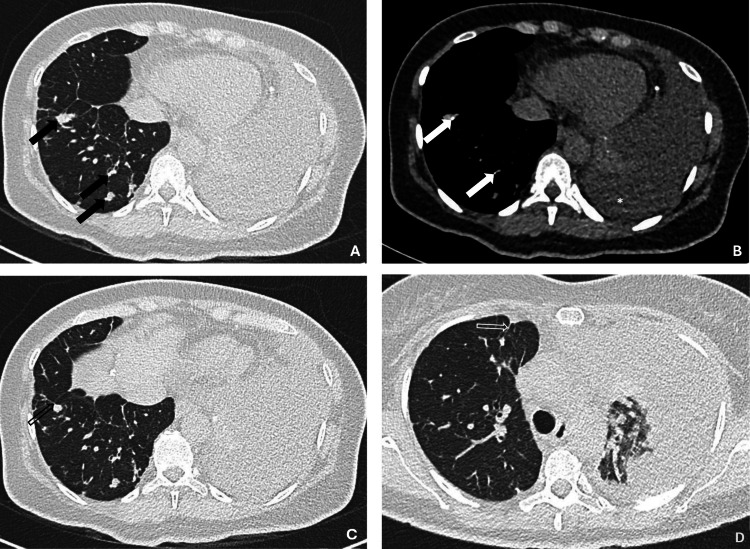
Axial CT images showing complex and mildly spiculated pulmonary nodules (A, black arrows) presenting punctuate calcifications (B, white arrows) and a large left pleural effusion (B, *). Note fissural involvement of pulmonary nodules (C, black open arrow) and the presence of pleural tags (D, white open arrow) CT: computed tomography

A chest X-ray showed a left pleural effusion, and a chest CT showed numerous additional irregular pulmonary nodules (Figure 1). These lesions had a median axial diameter of 5.9 mm (range 3.2 to 11.4 mm).

**Figure 2:**
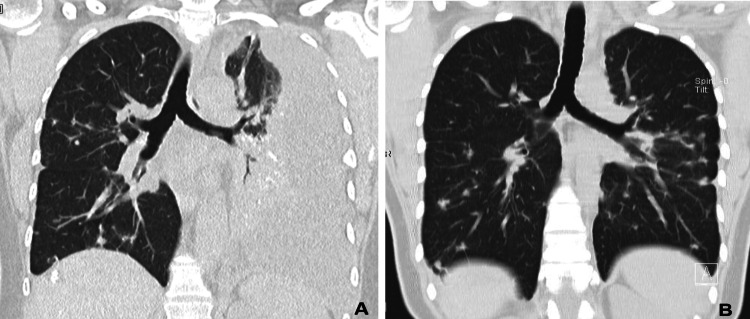
Coronal images of chest CT examinations at the carinal level at the time of current presentation to the emergency department (A) compared to a previous examination twenty years earlier (B) CT: computed tomography

For comparison, a thoracic CT scan examination performed 20 years earlier is shown in Figure 2.

Based on the pleuropulmonary manifestations in the CT study, differential diagnoses included benign and malignant etiologies such as Wegener granulomatosis, EHE, lymphoma, mesothelioma, epithelioid sarcoma, lung adenocarcinoma, and pleuropulmonary metastases. Since the patient had a history of EHE, the latter was the most probable diagnosis.

**Figure 3:**
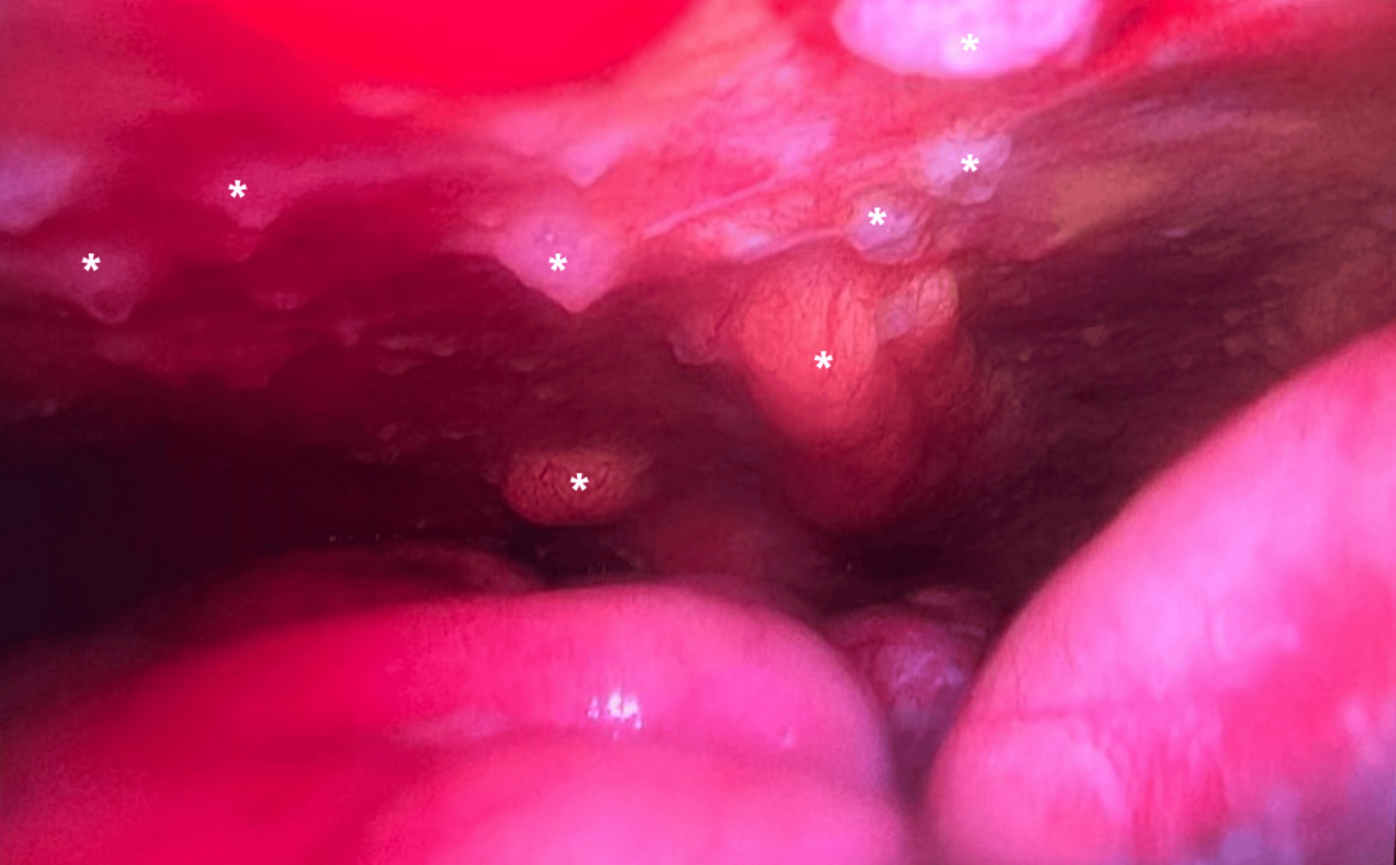
Intraoperative photo showing diffuse EHE nodular lesions within the parietal pleura (*) EHE: epithelioid hemangioendothelioma

A thoracocentesis was done in the days following admission. Bacteriology cultures were negative, and no neoplastic cells were observed within the fluid. A diagnostic thoracoscopy was then performed with biopsies of the pleura. Intraoperative examination showed diffuse nodular lesions within the parietal pleura (Figure 3).

**Figure 4:**
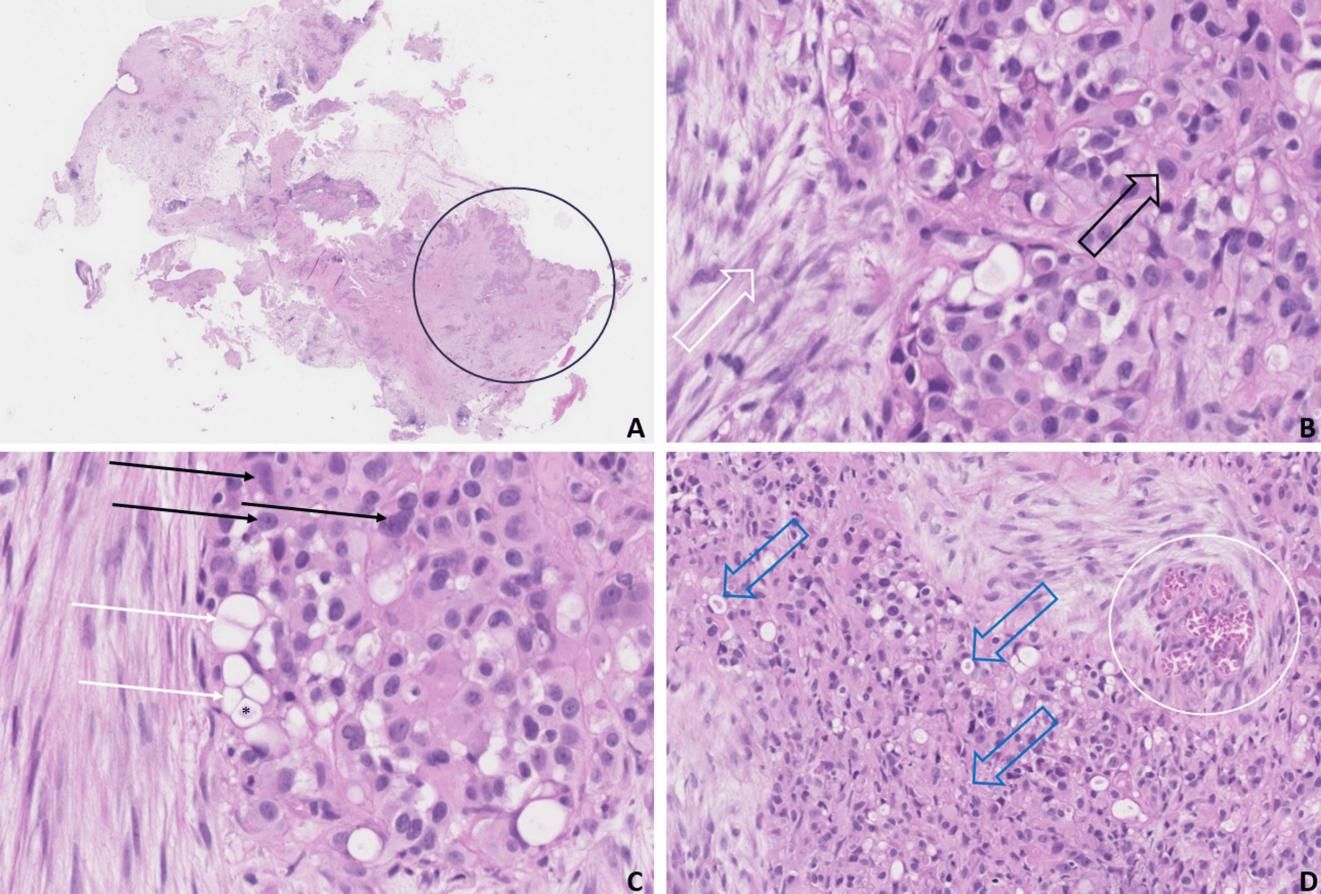
(A) Low-power examination of the pleural biopsy specimen, showing an ill-defined lesion (black circle). (B) High-power examination demonstrating fibrostromal reaction (white open arrow). Epithelioid cells (black open arrow) can also be visualized. (C) High-power examination showing cells exhibiting pleomorphism (black arrows). Note the presence of conglomerates of small abortive vascular structures (white arrows) manifesting with a pseudo-adipous aspect and wherein a red blood cell can be identified (*). (D) High-power examination showing a considerable amount of red blood cells (white circle) within the lumen of abortive vessels. Note the presence of dispersed hyaline globules (blue open arrows)

Biospsies of the nodular pleural lesions were submitted for histopathological examination (Figure 4). An extensive immunohistochemical panel showed diffuse proliferation of epithelioid cells with intense ERG+, CD34+, CD31+, and D2-40 markings. There was neither marked nuclear atypia nor marked mitotic activity. No tumor necrosis was observed. Moreover, a t(1;3)(p36.23;q25) WWTR1-CAMTA1 gene fusion was established upon sequencing. A fluorodeoxyglucose positron emission tomography CT scan was obtained, and no significant hypermetabolic lesion was observed.

Based on the investigations, the diagnosis of pleuropulmonary EHE was confirmed. Conservative management was decided at the multidisciplinary tour. The patient evolved well clinically at the six-month follow-up.

## Discussion

EHE is a rare vascular tumor, with a prevalence of 1 per 1,000,000 in the general population, that can affect diverse tissues [1,5]. Its clinical behavior varies between hemangioma and angiosarcoma, and prognosis varies depending on the affected organs [2]. In fact, some cases show atypical malignant features on specimen analyses, such as increased cytological atypia and increased mitotic activity with or without necrosis, and behave more aggressively [2]. None of these features was noted in our patient.

The etiology of this disease is not entirely understood [3]. However, several studies outline the role of CAMTA1 and WWTR1 gene rearrangements, which are found in over 90% of cases [1,3]. In fact, WWTR1::CAMTA1 fusion, which results from a t(1;3)(p36.3;q25) translocation, is considered a distinctive pathognomonic fusion gene along with the YAP1::TFE3 fusion, which is less commonly found [1,3].

A three-tiered risk assessment system for EHEs is proposed by Shibayama et al. based on tumor size (large tumor size defined as >30 mm) and histologic atypia (defined by having at least two of the following three findings: high mitotic activity, high nuclear grade, and coagulative necrosis) [5]. According to this stratification, low-risk, intermediate-risk, and high-risk groups showed five-year overall survival rates of 100%, 81.8%, and 16.9%, respectively [5]. In contrast, in this study, survival did not correlate with multifocality or multiorgan involvement [5].

The pleuropulmonary findings of the CT study in our patient are similar to those described in a case series by Liu et al. [6]. Those include punctuate calcifications and pleural indentation of pulmonary nodules [6]. Despite the fact that this disease lacks pathognomonic radiologic features, the joint presence of multiple pulmonary nodules with pleural indentation and punctate calcifications and pleural thickening should prompt the diagnosis of pleuropulmonary EHE [3].

There is no consensus for EHE treatment. Current clinical practice is based on retrospective case series or case reports [1]. Multiple treatment regimens have been described for pleuropulmonary EHE; the treatment of choice for confirmed unifocal EHE is surgical resection with or without adjuvant radiotherapy [1]. For locoregionally advanced EHEs or EHEs with systemic involvement, active surveillance is preferred [1].

## Conclusions

In this study, we provide detailed chest CT and thoracoscopy images of a 55-year-old woman diagnosed with pleuropulmonary EHE. Notably, her case exhibited no atypical histologic features. This case underscores the significance of conservative management for patients with this condition, as the absence of atypical features may suggest a less aggressive disease course, potentially reducing the need for more invasive treatments.

## References

[1] Stacchiotti S, Miah AB, Frezza AM (2021). Epithelioid hemangioendothelioma, an ultra-rare cancer: a consensus paper from the community of experts. ESMO Open.

[2] Fan Y, Wang F, Li S, Ye C, Ying Y, Mao H (2016). Pleural epithelioid hemangioendothelioma: a case report and literature review. J Natl Med Assoc.

[3] Abdelmogod A, Papadopoulos L, Riordan S (2023). A matched molecular and clinical analysis of the epithelioid haemangioendothelioma cohort in the Stafford Fox rare cancer program and contextual literature review. Cancers (Basel).

[4] World Health Organization (2002). Pathology and genetics of tumours of soft tissue and bone.

[5] Shibayama T, Makise N, Motoi T (2021). Clinicopathologic characterization of epithelioid hemangioendothelioma in a series of 62 cases: a proposal of risk stratification and identification of a synaptophysin-positive aggressive subset. Am J Surg Pathol.

[6] Liu K, Xie P, Peng W, Zhou Z (2014). The computed tomographic findings of pulmonary epithelioid hemangioendothelioma. Radiol Med.

